# Incidence, risk and prognosis of second primary malignancy of patients with gastric adenocarcinoma

**DOI:** 10.1038/s41598-024-56408-4

**Published:** 2024-03-08

**Authors:** Liyan Jin, Xinyue Su, Wenjing Li, Jie Wu, Hua Zhang

**Affiliations:** 1 Department of Oncology, Wujin Hospital Affiliated with Jiangsu University, Yong Ning North Road No. 2, Changzhou, 213000 Jiangsu China; 2 Department of Oncology, The Wujin Clinical College of Xuzhou Medical University, Yong Ning North Road No. 2, Changzhou, 213000 Jiangsu China; 3 Department of Public Course, Jiangsu College of Nursing, Science Road No. 9, Huai’an, 210023 Jiangsu China

**Keywords:** Gastric cancer, Second primary malignancy, SEER, Survival analysis, Cancer, Cancer, Epidemiology

## Abstract

Due to the long-term low survival rates of gastric adenocarcinoma (GAC) patients, the occurrence and prognosis of second primary malignancies (SPMs) are often underreported and overlooked as a significant concern.To date, only a few studies have addressed this issue in the context of GAC. These studies, however, are limited by their small patient cohorts and lack of substantial, meaningful findings. Our study aims to fill this gap by investigating the incidence, risk factors, and prognostic significance of SPMs among GAC survivors. Utilizing the Surveillance, Epidemiology, and End Results (SEER) database, we analysed data from patients diagnosed with GAC between 2000 and 2020. The study employs the standardized incidence ratio (SIR) to assess the relative risk of SPMs, competing risk regression to identify risk factors for SPM development after GAC, and Kaplan-Meier and COX regression analyses for survival outcomes. Out of 44,041 GAC patients analyzed, 2,032 (4.3%) developed SPMs, with a median latency period of 36 months. The incidence of SPMs was significantly higher in GAC patients (SIR 1.36, 95% CI 1.32-1.4, EAR 53.57) compared to the general population. Key factors including older age, sex, tumor grade, summary stage, and history of surgical and radiation therapy were related to the higher risk of developing SPMs following GAC. Interestingly, GAC patients without SPMs exhibited poorer overall survival compared to those with SPMs. Age, summary stage, and surgical history were identified as independent prognostic factors for GAC patients with SPMs. This comprehensive analysis underscores the necessity of vigilant monitoring and tailored follow-up for SPMs in GAC survivors, highlighting the study's contribution to enhancing GAC survivors care strategies.

## Introduction

Gastric cancer (GC) represents a significant burden in global cancer-related mortality, accounting for over 700,000 deaths annually^1^. Adenocarcinoma, the most prevalent subtype, accounts for more than 90% of GC cases^2^. The prognosis of tumor has improved remarkably due to advancements in early diagnosis, treatment and surveillance^3^. However, long-term survivors still encounter various challenges, including physical, psychosocial, medical, behavioral, and socioeconomic consequences of cancer and its treatment^4^. Notably, the increased probability of a subsequent diagnosis of another cancer poses a great threat to their lives, necessitating urgent investigation into this issue^5,6^. A surge of recent studies has investigated the incidence, risk factors, and survival of patients with a second primary malignancies (SPMs) following various cancers, such as colorectal^7^, lung^8^, minor salivary gland^9^, and ovarian clear cell carcinoma^10^.

Due to the long-term low survival rates of gastric adenocarcinoma (GAC) patients, the occurrence and prognosis of SPMs are often underreported and overlooked as a significant concern. To date, only a few studies have addressed this issue in the context of GAC. These studies, however, are limited by their small patient cohorts and lack of substantial, meaningful findings. For example, Zheng^11^ and Wang’s research^12^ merely outlined the clinicopathologic characteristics of SPMs in GC, Shah et al.^13^ identified an increased risk of SPMs in GC patients compared to the general population, and Kim’s study^14^ evaluated the risk of developing multiple primary malignancies (MPMs) in a sample of 3066 patients who had undergone curative resection of GC. This underscores the critical need for more extensive, large-scale research into the incidence, risk factors, and prognosis of SPMs in GAC patients.

In this study, utilizing the Surveillance, Epidemiology, and End Results (SEER) research database, we aimed to assess the incidence of SPMs in GAC patients, identify risk factors associated with developing SPMs following GAC, and explored the prognostic factors of SPMs in GAC patients. Concurrently, we present the characteristics of Chinese GAC patients with SPMs from a single center. This research could potentially guide the establishment of more effective strategies and preventative measures for post-treatment surveillance in GAC cases.

## Material and methods

### Data source

Dataset of gastric adenocarcinoma were obtained from SEER Research Plus Data, 17 Registries, Nov 2022 Sub (2000–2020) in the Surveillance, Epidemiology, and End Results (SEER) database (http://seer.cancer.gov), covering approximately 26.5% of the U.S. population. The multiple primary standardized incidence ratio (MP-SIR) session and Case Listing Session of SEER *Stat software version 8.4.2 (Surveillance, Research Program, National Cancer Institute, Bethesda, MD) were used to extract the detailed demographic and characteristic data, including age at diagnosis, sex, sequence number, site, summary stage, treatment information and survival months. The ethics approval was not required since SEER databases were anonymized publicly available.

### Data collection

**Figure 1:**
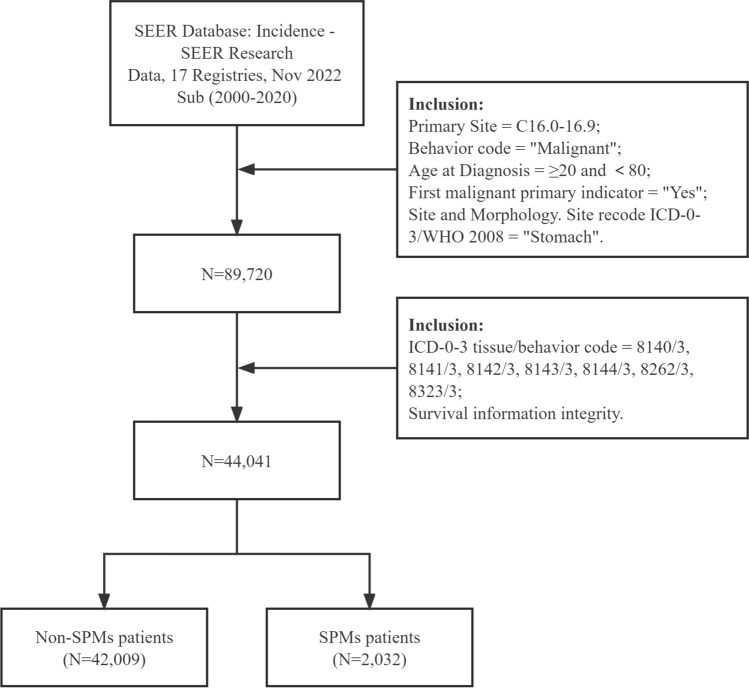
The flow chart of the screening process for GAC patients. GAC, gastric adenocarcinoma.

Patients diagnosed with a first primary gastric adenocarcinoma aged from 20 to 80 years between 2000 and 2020 were retrieved, with their tumor site was restricted as stomach cancer (C16.0–C16.9) according to Third Edition of International Classification of Diseases for Oncology (ICD-O-3) and first malignant primary indicator code “Yes”, behavior code “Malignant”. To further focus to patients with adenocarcinoma, we included patients with ICD-O-3 tissue/behavior codes 8140/3, 8141/3, 8142/3, 8143/3, 8144/3, 8262/3 and 8323/3. Finally, the patients were excluded if meeting the exclusion criteria as follow: (1) latency period of fewer than 2 months between initial primary malignancy (IPM) and SPMs^15^; (2) uncertain follow-up time and latency period. SPMs were defined as second malignancy according to key clinical information on “malignant tumors for patient” and the “sequence number” of the multiple primary malignancies. Clinicopathological information was gathered including age, sex, race, summery stage, grade, treatment characteristics, survival months and status. A flowchart displaying the detailed selection process is presented in Fig. 1.

#### Real-world patients data

Real-world study included patients diagnosed with SPMs following initial gastric adenocarcinoma diagnoses, treated at Jiangsu University's Wujin Hospital Department of Oncology. The cohort was gathered between May 2012 and June 2022 and staged based on the AJCC TNM Cancer Staging Manual, Sixth Edition.

### Statistical analysis

The chi-square test or exact Fisher’s test was employed for categorical variables, while the Wilcoxon Mann–Whitney U-test was used for continuous variables to contrast the differences between OPM and SPM groups. The MP-SIR algorithm of the Seer*stat program was used to obtain standardized incidence ratios (SIR) and excess absolute risk (EAR) for second primary malignancies in patients with first primary gastric adenocarcinoma compared to a reference group representative of the general population. The cumulative incidence of SPMs development was assessed by using Fine and Gray’s competing risk regression, accounting for competing risk of death and non-SPMs. Furthermore, the multivariable risk regression based on proportional subdistribution hazard model was performed included the covariates with variables with 2-sided *p* < 0.05 in univariable analysis. The Kaplan–Meier method and log-rank test were used for the survival analysis. A Cox proportional hazards regression model was used for the univariate and multivariate analyses to identify prognostic factors in SPMs patients. The SPSS v25.0 (IBM, Armonk, NY, USA) and R for Windows v4.1.0 (https://www.r-project.org) were used for the statistical analysis. All tests were two-way and *p* < 0.05 was considered statistically significant.

### Ethics approval and consent to participate

No additional informed consent was required for the anonymized and de-identified data from the SEER database. The study was complied with the 1964 Helsinki Declaration and its later amendments or comparable ethical standards. The study was approved by the ethics committee of Wujin Hospital Affiliated with Jiangsu University. Informed consent was obtained from all patients and/or their legal guardian(s).

## Results

### Characteristics of patients

The study group final remained 44,041 identified patients, including 31,254 male (71.0%) and 12,787 female individuals (29.0%). Also, it consisted of 22,920 (52.0%) over 65 years old, 31,170 (70.8%) white, 22,235 (50.5%) classified as Grade III and 18,364 (41.7%) classified as distant stage. As for treatment, 20,439 (46.4%) patients were under surgery, 12,945 (29.4%) were under radiation and 24,587 (55.8%) were under chemotherapy. At the end of follow-up, 2,032 of all patients (4.3%) were reported SPM occurrence. The median latency time of SPMs was 36 months (interquartile range, 15–75 months). In comparison with none SPMs patients, those with SPMs contained more older (≥ 65 years), more male, more percentage of low grade and summary stage. The baseline characteristics of all patients were listed in Table 1.

**Table 1:** Clinical characteristics of patients in the study.

Variables	Overall(n = 47,651)	SPMs incurrence		
		None SPMs (n = 42,009)	With SPMs (n = 2032)	*P*-value
Age				< 0.001
< 65	21,121 (48.0%)	20,282 (48.3%)	839 (41.3%)	
≥ 65	22,920 (52.0)	21,727 (51.7%)	1193 (58.7%)	
Sex				0.001
Male	31,254 (71.0%)	29,745 (70.8%)	1509 (74.3%)	
Female	12,787 (29.0%)	12,264 (29.2%)	523 (25.7%)	
Race				0.254
White	31,170 (70.8%)	29,744 (70.8%)	1426 (70.2%)	
Black	5753 (13.1%)	5487 (13.1%)	266 (13.1%)	
AI/API	6891 (15.6%)	6556 (15.6%)	335 (16.5%)	
Unknown	227 (0.5%)	222 (0.5%)	5 (0.2%)	
Grade				< 0.001
Grade I	1883 (4.3%)	1722 (4.1%)	161 (7.9%)	
Grade II	12,598 (28.6%)	11,827 (28.2%)	771 (37.9%)	
Grade III	22,235 (50.5%)	21,377 (50.9%)	858 (42.2%)	
Grade IV	2276 (5.2%)	2229 (5.3%)	47 (2.3%)	
Unknown	5049 (11.5%)	4854 (11.6%)	195 (9.6%)	
Summary stage				< 0.001
Localized	8757 (19.9%)	7862 (18.7%)	895 (44.0%)	
Regional	14,251 (32.4%)	13,399 (31.9%)	852 (41.9%)	
Distant	18,364 (41.7%)	18,175 (43.3%)	189 (9.3%)	
Unknown	2669 (6.1%)	2573 (6.1%)	96 (4.7%)	
Surgery				< 0.001
Yes	20,439 (46.4%)	18,795 (44.7%)	1644 (80.9%)	
No	23,602 (53.6)	23,214 (55.3%)	388 (19.1%)	
Radiation				< 0.001
Yes	12,945 (29.4%)	12,140 (28.9%)	805 (39.6%)	
None/Unknown	31,096 (70.6%)	29,869 (71.1%)	1227 (60.4%)	
Chemotherapy				0.038
Yes	24,587 (55.8%)	23,498 (55.9%)	1089 (53.6%)	
None/unknown	19,454 (44.2%)	18,511 (44.1%)	943 (46.4%)	

### SPM incidence

As shown in Table 2, the incidence of total malignancies was higher than that of the general population (SIR 1.36, 95% CI 1.32 to 1.4, EAR 53.57). And the increased incidence of SPMs was found in the 2–11 months (SIR 1.28, 95% CI 1.19 to 1.37, EAR 38.25), 12–59 months (SIR 1.48, 95% CI 1.41 to 1.54, EAR 68.82), 60–119 months (SIR 1.27, 95% CI 1.2 to 1.34, EAR 42.05) and over 120 months (SIR 1.28, 95% CI 1.19 to 1.38, EAR 47.66). Then, we found that the most common sites for SPMs were Stomach (17.4%), Prostate (12.6%), Lung and Bronchus (12.1%), Breast (6.5%), Urinary Bladder and Kidney (4.5%), Pancreas (4.2%) and Kidney (3.8%) (Supplementary Table 1). Meanwhile, patients with gastric adenocarcinoma had different risk of SPMs (Table 3) with site-specific differences based on the above main SPMs sites. Patients with gastric adenocarcinoma had increased risk of stomach (SIR 12.68, 95% CI 11.86 to 13.55, EAR 33.39), pancreas (SIR 1.77, 95% CI 1.59 to 2.04, EAR 3.65), kidney (SIR 1.6, 95% CI 1.38 to 1.85, EAR 2.96) and lung and bronchus (SIR 1.19, 95% CI 1.1 to 1.29, EAR 4.05); conversely, they exhibited decreased risk of prostate (SIR 0.83, 95% CI 0.77 to 0.91, EAR -4.73) and breast (SIR 0.88, 95% CI 0.79 to 0.99, EAR -1.64).

**Table 2:** Standardized incidence ratios and excess absolute risks of secondary malignancy distributed by time from diagnosis of the primary gastric adenocarcinoma.

All Sites	2–11 months	12–59 months	60–119 months	120 + months	Total
Observed	816	2229	1196	683	4924
SIR	1.28	1.48	1.27	1.28	1.36
95% CI Lower	1.19	1.41	1.2	1.19	1.32
95% CI Upper	1.37	1.54	1.34	1.38	1.4
EAR	38.25	68.82	42.05	47.66	53.57

SIR, Standardized incidence ratio; EAR, Excess absolute risk is per 10,000.

**Table 3:** Standardized incidence ratios and excess absolute risks of secondary malignancy distributed by the main sites following the first primary gastric adenocarcinoma.

	Total					
	Observed	Expected	SIR	CI Lower	CI Upper	EAR
All Sites	4924	3624.69	1.36	1.32	1.4	53.57
Stomach	879	69.31	12.68	11.86	13.55	33.39
Prostate	578	692.73	0.83	0.77	0.91	− 4.73
Lung and Bronchus	618	519.67	1.19	1.1	1.29	4.05
Breast	304	343.8	0.88	0.79	0.99	− 1.64
Urinary Bladder	217	196.9	1.1	0.96	1.26	0.83
Pancreas	203	114.42	1.77	1.54	2.04	3.65
Kidney	191	119.2	1.6	1.38	1.85	2.96

SIR, Standardized incidence ratio; EAR, Excess absolute risk is per 10,000.

### Risk factors of developing SPMs

The results of risk factor of developing SPMs after gastric adenocarcinoma are shown in Table 4. In multivariable competing risk analysis, the higher risk of SPMs was associated significantly with older patients (≥ 65 years: HR 1.207, 95% CI 1.104–1.320, *p* < 0.001), while female patients (HR 0.850, 95% CI 0.769–0.940, *p* < 0.01), higher-grade patients (Grade III: HR 0.765, 95% CI 0.643–0.909, *p* < 0.01; Grade IV: HR 0.641, 95% CI 0.462–0.890, *p* < 0.01), advanced-stage patients (Regional: HR 0.534, 95% CI 0.483–0.590, *p* < 0.001; Distant: HR 0.190, 95% CI 0.159–0.227, *p* < 0.001), non-surgical patients (HR 0.377, 95% CI 0.331–0.430, *p* < 0.001) and patients without radiotherapy (HR 0.699, 95% CI 0.635–0.769, *p* < 0.001) had a lower risk of developing SPMs after GAC.

**Table 4:** Univariable and multivariable competing risk regression analysis of risk of developing second primary malignancies.

Variables	Univariable analysis		Multivariable analysis	
	HR (95% CI)	*P*-value	HR (95% CI)	*P*-value
Age				
< 65	Reference		Reference	
≥ 65	1.310 (1.200–1.430)	< 0.001	1.207 (1.104–1.320)	< 0.001
Sex				
Male	Reference		Reference	
Female	0.846 (0.767–0.935)	< 0.001	0.850 (0.769–0.940)	< 0.01
Race				
White	Reference		Reference	
Black	1.005 (0.882–1.140)	0.94		
AI/API	1.069 (0.950–1.200)	0.27		
Unknown	0.726 (0.301–1.750)	0.48		
Grade				
Grade I	Reference		Reference	
Grade II	0.716 (0.605–0.848)	< 0.001	0.947 (0.797–1.124)	0.53
Grade III	0.449 (0.380–0.532)	< 0.001	0.765 (0.643–0.909)	< 0.01
Grade IV	0.342 (0.247–0.473)	< 0.001	0.641 (0.462–0.890)	< 0.01
Unknown	0.435 (0.353–0.536)	< 0.001	0.991 (0.801–1.225)	0.93
Summary stage				
Localized	Reference		Reference	
Regional	0.572 (0.521–0.628)	< 0.001	0.534 (0.483–0.590)	< 0.001
Distant	0.098 (0.084–0.115)	< 0.001	0.190 (0.159–0.227)	< 0.001
Unknown	0.356 (0.289–0.439)	< 0.001	0.641 (0.515–0.799)	< 0.001
Surgery				
Yes	Reference		Reference	
No	0.206 (0.185–0.230)	< 0.001	0.377 (0.331–0.430)	< 0.001
Radiation				
Yes	Reference		Reference	
None/unknown	0.653 (0.598–0.714)	< 0.001	0.699 (0.635–0.769)	< 0.001
Chemotherapy				
Yes	Reference			
None/unknown	1.060 (0.976–1.160)	0.16		

### Survival of SPMs

**Figure 2:**
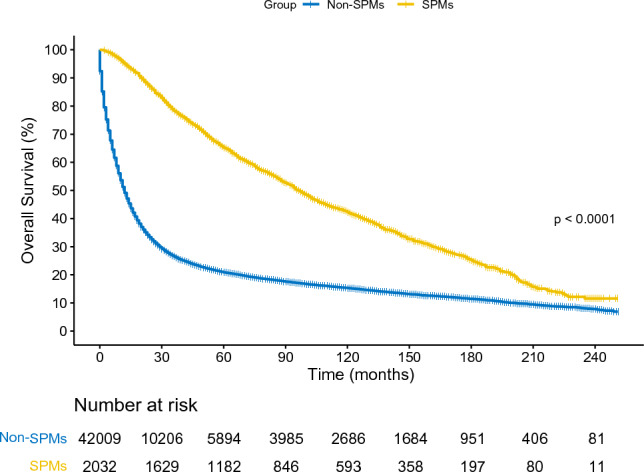
Kaplan–Meier curves of GAC patients with and without SPMs. GAC, gastric adenocarcinoma.

The Kaplan–Meier survival curves demonstrated that patients with SPMs had a significantly improved overall survival compared to those non-SPMs patients (Fig. 2, log-rank *p* < 0.001). Then, univariable and multivariable Cox regression analysis was applied to reveal OS-related factors in SPMs. The results (Table 5) show that age (*p* < 0.001), summary stage (Regional: *p* < 0.001; Distant: *p* < 0.001) and surgical history (*p* < 0.001) were independent predictive variables for SPMs survival.

**Table 5:** Univariable and multivariable COX analysis of overall survival in second primary malignancies patients.

Variables	Univariable analysis		Multivariable analysis	
	HR (95% CI)	*P*-value	HR (95% CI)	*P*-value
Age				
< 65	Reference		Reference	
≥ 65	1.427 (1.274–1.597)	< 0.001	1.516 (1.353–1.699)	< 0.001
Sex				
Male	Reference			
Female	1.030 (0.908–1.168)	0.646		
Race				
White	Reference			
Black	1.094 (0.893–1.340)	0.386		
AI/API	0.555 (0.078–3.960)	0.557		
Unknown	1.205 (1.038–1.400)	0.014		
Grade				
Grade I	Reference			
Grade II	1.056 (0.843–1.322)	0.635		
Grade III	1.272 (1.019–1.588)	0.034		
Grade IV	1.533 (0.934–2.516)	0.091		
Unknown	1.115 (0.848–1.466)	0.436		
Summary stage				
Localized	Reference		Reference	
Regional	1.295 (1.150–1.459)	< 0.001	1.393 (1.235–1.571)	< 0.001
Distant	2.541 (2.114–3.054)	< 0.001	1.730 (1.407–2.129)	< 0.001
Unknown	1.384 (1.069–1.791)	0.014	0.913 (0.693–1.204)	0.519
Surgery				
Yes	Reference		Reference	
No	2.330 (2.041–2.660)	< 0.001	2.258 (1.925–2.650)	< 0.001
Radiation				
Yes	Reference			
None/Unknown	0.882 (0.790–0.984)	0.025		
Chemotherapy				
Yes	Reference			
None/Unknown	0.793 (0.711–0.885)	< 0.001		

### Characteristics of GAC patients with SPMs in eastern China

The clinicopathologic characteristics of the 41 GAC patients treated at Wujin Hospital were summarized in Table 6. The average age of the patients was 68.66 years, with 33 out of the 41 (80.5%) being male. In terms of TNM staging, By the end of the follow-up period, 23 patients (56.1%) were alive. The four most common sites for the development of SPMs were the esophagus, rectal, colon, and prostate. Alternatively, the median OS of these patients was 49 months, with a 5-year survival rate of 54.4%.

**Table 6:** Clinicopathological characteristics of second primary malignancies patients following gastric adenocarcinoma in Wujin Hospital.

Variable	No. of patients (%)
Age (year, mean ± SD)	68.66 ± 6.07
Sex	
Male	33(80.5)
Female	8(19.5)
TNM stage	
I	18(43.9)
II	8(19.5)
III	9(22.0)
IV	6(14.6)
Site of SPMs	
Esophagus	17(41.5)
Rectal	9(22.0)
Colon	8(19.5)
Prostate	3(7.3)
Lung	1(2.4)
Bladder	1(2.4)
Liver	1(2.4)
Appendix	1(2.4)
Status	
Dead	18(43.9)
Alive	23(56.1)

## Discussion

To the best of our knowledge, this study represents the most extensive population-based analysis of the incidence and prognosis of SPMs following GAC. Based on SEER database, we analyzed total 44,041 patients with GAC, 2,032 (4.61%) of them developed SPMs, and found an elevated incidence of SPMs compared to the general population. Moreover, our study evaluated the risk factors associated with the occurrence of SPMs and the survival outcomes of SPMs after GAC.

The rising number of cancer survivors worldwide annually has made SPMs an increasingly significant threat to health. Previous studies have reported global SPMs incidence rates for GC patients ranging from 4.4 to 5.5% worldwide^16^. In our study, we identified 4.61% of GAC patients developed SPMs following the initial diagnosis. Further, Zheng et al.^11^ observed a median duration of 46.9 months from the initial diagnosis to the emergence of SPM, in contrast to the 36 months median time reported in our study. The incidence of SPMs among GC survivors is notably higher compared to the general population. This disparity may stem from the therapeutic approaches used for primary caners, genetic predispositions and shared environmental factors^17^. Shah’s study^13^ revealed a 1.06 to 1.16-fold increase in the risk of developing SPMs in GC cases between from 1992 to 2012. In contrast, our findings indicate a more pronounced risk ratio in GAC between 2000 and 2020, possibly due to variations in histology and the evolution of treatment approaches. Additionally, our study identified an elevated SIR within a latency period of 12–59 months, paralleling Shah’s findings where the median interval from initial diagnosis to development of the first SPM was 46.9 months^13^. We also found that the most common sites for SPMs in GAC are the stomach, prostate, lung and bronchus, while another US population-based study identified the stomach, small intestine, and esophagus as the predominant sites^13^. A population study in Japan revealed that the thyroid, esophagus, and mouth/pharynx are the three most common sites for SPMs in all cancer patients between 1985 and 2007^18^. Conversely, in Taiwan, the leading sites were non-Hodgkin's lymphoma, ovaries, and the esophagus in SPMs patients after GC^19^. Contrarily, smaller-scale studies often point to the digestive tract, including the esophagus, small bowel, and colon, as the frequent sites for SPMs^16,20^. Such incidence patterns in GAC highlight the critical need for enhanced surveillance and regular endoscopic examinations to effectively manage these cases.

The underlying mechanism leading to the development of SPMs after GC remain largely unclear. Factors such as genetic susceptibility, immunological aspects, and exposure to carcinogens, including those used in GC treatments, are deemed significant^21,22^. Our results suggested that age, sex, tumor grade, summary stage, and histories of surgery and radiation therapy are independent risk factors of developing SPMs following GAC. Chen et al.^19^ reported that being male, having diabetes mellitus, COPD, and liver cirrhosis, along with being 70 years or older, were independent predictors for the development of SPMs in GC patients, as determined by Multivariate Cox proportional hazards analysis. Morais et al.^23^ suggested that pre-diagnosis lifestyles might affect the occurrence of an SPM among GC survivors in the long term. Moreover, treatments such as chemotherapy^24^, radiotherapy^25^, and surgery^26^ have been implicated in the development of SPMs. Nevertheless, additional research is imperative to further substantiate these risk factors for SPMs following GAC.

Due to their rarity, few studies have analyzed the survival outcomes of patients with SPMs following their initial primary cancer. In our research, we observed that patients without SPMs exhibited poorer overall survival compared to those with SPMs. This finding contrasts with the observation from Kim’s study^14^, which reported that the 5-year survival rates of GC patients with SPMs were statistically lower than those without SPMs, as determined through single-institutional retrospective research. Concurrently, the prognosis differed between SPMs and non-SPMs patients in various types of cancer. In hepatocellular carcinoma, notable survival differences were observed between the two groups^27^. In contrast, no significant survival disparities were found in cases of ovarian clear cell carcinoma^10^ and malignancies of the eye and ocular adnexa^28^. SPM group patients in our study, characterized by lower pathological grades and earlier stages at diagnosis, receive more frequent follow-ups. This approach likely contributes to their improved prognosis, but further research is needed to explore the reasons affecting the prognosis of different types of cancer patients with SPM in a more comprehensive and in-depth manner. Furthermore, our study indicated that age, summary stage and surgical history were independent prognosis factors for SPMs patients, and the prognosis of Chinese patients was worse than that of U.S. patients. This discrepancy may be influenced by differences in race, etiologies, and treatment strategies. Ha et al. revealed that the 5-year survival rates of stage I, II, and III GC patients with SPMs in Korea were 61%, 39%, and 30%, respectively^29^. Although only 41 cases of Chinese SPM gastric adenocarcinoma patients were included in this study, compared with the previous study of 78 Chinese SPM gastric cancer patients^12^, the esophagus is still the most common site for SPM. However, the sequence of other common SPM sites shows slight variations. As expected, a more comprehensive global analysis was required to fully understand the prognosis of GC patients with SPMs.

This study has several limitations. First, key characteristics, such as tobacco use, alcohol consumption, obesity, and family history of cancer, were unavailable in the SEER database. Second, the inherent limitations of the SEER database may affect the reliability of our conclusions, particularly in distinguishing between second primary cancers and tumor recurrences. Third, detailed therapy information, such as radiotherapy dosage and adjuvant chemotherapy, which may be associated with the development of SPMs, was not accessible. Lastly, the availability of real-world data pertaining to Chinese patients was limited.

## Conclusions

This population-based study demonstrated an increased incidence of SPMs among GAC survivors compared to the general population. Key independent risk factors for developing SPMs following GAC included age, sex, tumor grade, summary stage, and history of surgical and radiation therapy. Additionally, age, summary stage, and surgical history emerged as independent prognostic factors for GAC patients with SPMs. Therefore, post-treatment surveillance in GC should be considered during follow-up not only to detect recurrence but also to ensure the early identification of SPMs, and further research is needed to understand the mechanisms underlying the development of SPMs.

### Supplementary Information

Supplementary Table 1.

## Data Availability

The data is available in the Surveillance, Epidemiology, and End Results database (https://seer.cancer.gov).

## Abbreviations

GC: Gastric cancer
SPMs: Second primary malignancies
MPMs: Multiple primary malignancies
SEER: The surveillance, epidemiology, and end results
SIR: Standardized incidence ratio
